# Polyarticular arthritis as presenting feature of farber disease: a lysosomal storage disease involving inflammation

**DOI:** 10.1186/1546-0096-12-S1-P285

**Published:** 2014-09-17

**Authors:** Alexander Sólyom, Nesrin Karabul, Boris Hügle, Calogera Simonaro, Edward Schuchman

**Affiliations:** 1 Villa Metabolica, Children's Hospital, University Medical Center, Mainz, Germany; 2 German Center for Pediatric and Adolescent Rheumatology, Garmisch-Partenkirchen, Germany; 3 Genetics and Genomic Sciences, Mt. Sinai School of Medicine, New York, USA

## Introduction

Farber lipogranulomatosis (Farber Disease; FD) is an ultra-rare lysosomal storage disorder resulting from the inherited deficiency of the enzyme acid ceramidase, and the accumulation of the lipid substrate, ceramide. Ceramide is a pro-inflammatory and pro-apoptotic lipid that has been implicated in the pathogenesis of cartilage disorders. Farber Disease has a heterogeneous presentation ranging from a severe phenotype with respiratory and CNS involvement with an average life expectancy of 1.3 years, to a moderate phenotype, which generally includes joint swelling, contractures and pain. The clinical similarity between the moderate Farber phenotype and the more severe forms of Juvenile Idiopathic Arthritis (JIA) suggests that moderate Farber Disease cases may be diagnosed as JIA in some cases.

## Objectives

To demonstrate the presentation, phenotype and pathophysiology of Farber Disease, and to show that moderate Farber Disease should be considered in certain cases of early-onset polyarticular arthritis in children. In addition, we address how the diagnosis of Farber Disease is established and the potential effectiveness of enzyme replacement therapy.

## Methods

We present a case study to demonstrate the moderate Farber phenotype and the results of a literature search of Farber Disease case studies since 1990. The background for enzyme replacement therapy (enzyme uptake and decrease of ceramide levels in vitro) also is outlined.

## Results

The patient described was originally diagnosed with JIA. The clinical suspicion of Farber Disease was raised by a pediatric rheumatologist with experience in lysosomal storage diseases. His leukocyte acid ceramidase activity is ~4% of his parents, and his ceramide levels are ~2 times that of his parents. Genetic testing revealed a homozygous mutation in the *ASAH1* gene. He is currently 9 years old and receiving anti-inflammatory therapy including a TNF-alpha blocker. He has no CNS involvement, but his joint disease continues to progress. Of the published Farber Disease case studies since 1990, 40% (n=14) described patients with moderate disease. 36% (n=5) of these patients were initially diagnosed as having JIA. To establish proof-of-concept for enzyme replacement therapy of Farber Disease, recombinant human acid ceramidase was produced in Chinese hamster ovary cells and added to the culture media of human Farber cells, resulting in a significant reduction in ceramide levels.

## Conclusion

A high percentage of moderate Farber Disease patients are likely initially suspected of having JIA. Given this finding, it is important to increase awareness of Farber Disease in the pediatric rheumatology community. Differential diagnosis can be made by accounting for the comparatively early onset, progressive symmetric arthritis, presence of subcutaneous nodules in the joints, scalp and/or spine, and an unusual, hoarse cry or voice (due to nodule formation on the larynx) in Farber Disease patients. Based on experience with Farber Disease cells and with bone marrow transplantation in Farber Disease patients, enzyme replacement therapy could reduce ceramide levels and resolve or significantly attenuate the arthritis phenotype in patients. Such therapy is currently under development. We propose that Farber Disease can account for specific cases diagnosed as JIA, and that clinically guided screening of the JIA population will reveal patients who will benefit from disease-specific care and treatment.

## Disclosure of interest

A. Sólyom Consultant for: Plexcera Therapeutics LLC, N. Karabul: None declared, B. Hügle: None declared, C. Simonaro: None declared, E. Schuchman Shareholder of: Plexcera Therapeutics LLC.

